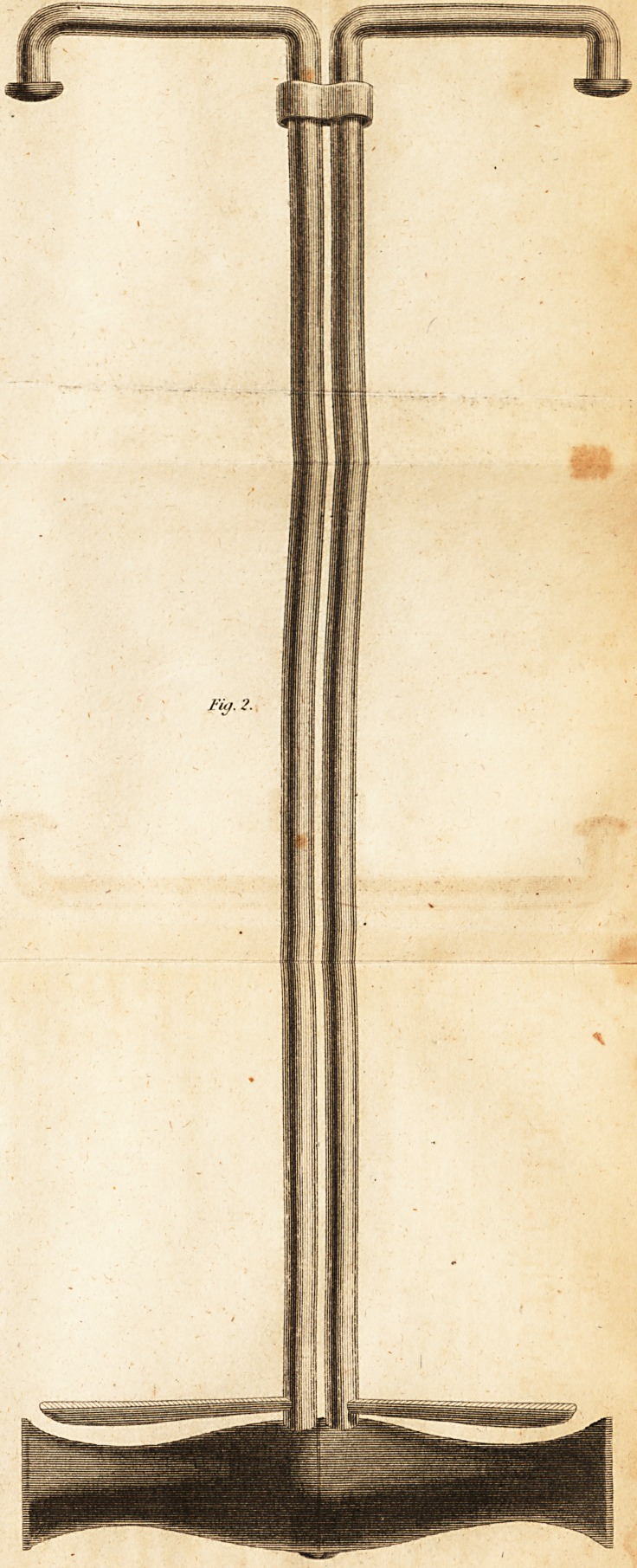# Mr. Guy's Instrument for Extracting the Fœtus

**Published:** 1809-08

**Authors:** William Guy

**Affiliations:** Chichester


### *To the Editors of the Medical and Physical Journal.*

GENTLEMEN,

IN the course of five and thirty years extensive practice in midwifery, I have, in three or four instances, been obliged to have recourse to one of the most painful operations that can possibly occur to a feeling mind; namely, opening the head of the child. An act, at which, as has been truly observed, human nature revolts. Still, however, it sometimes becomes a necessary duty; and, as I flatter myself, by making a less extensive opening into the cranium than has been usually recommended, material advantage when in the act of extracting will be derived, I wish, through the medium of your very useful Journal, to offer a few remarks on the subject.

By perforating the head, the surgeon has evidently two objects in view; the one, to evacuate the brain, and thus to afford a greater facility for the bones of the cranium to collapse; the other, to obtain a firm and fixed point, on which he may safely exert that degree of force, which may be requisite for the extraction of the fœtus. But I am inclined to think, that too great stress has been laid on the necessity of the former object; and I much doubt, whether the supposed advantages derived from the removal of the brain, are sufficient to counterbalance the inconvenience, which, when we come to extract, must necessarily occur, from the free and extensive opening generally directed. The brain, from its soft texture and compressibility, cannot afford any material resistance to the bones collapsing; this fact almost daily experience evinces. How frequently do we find, in long and severe labours, that they will collapse considerably, that the head will become elongated; and this, without detriment to the child. I am ready to admit, on the contrary, that in many cases, equally tedious and severe, the head will not collapse, but remains sound, compact, and firmly locked in the pelvis; but this difference in the state of the latter case evidently arises, not from the resistance of the brain, but from the extraordinary ossification of the bones, and from the sutures being more perfectly fectly formed, which prevents them from yielding, as the head is forced into the pelvis. In such cases, were all the brain evacuated, little or no collapse would ensue.

Supposing the above remarks well founded, it surely must be unnecessary to make a larger opening into the cranium, than will be sufficient easily to admit the extracting instrument; namely, about the size of a shilling. Hence, when we begin to extract, a much greater security will be afforded to our exertions, than if the cranium had been more freely divided; for, in such cases, even in the hands of the most expert, and of course conducted with the most guarded care, the instrument will be very apt to lose its hold and slip out, or the bone suddenly to give way, to the no small danger of the mother.

With respect to the instruments generally used for extracting the fœtus, I confess I am somewhat dissatisfied; for, whether the blunt hook or crotchet be employed, from the small distance from the edge of the bone, on which the curvature of either will admit their point to be fixed, they will frequently slip out. To obviate this inconvenience, I beg leave to recommend an instrument which I trust will be found to possess the following advantages.

First. When closed, in which case the two blunt ends are in contact, they may be introduced into a small opening with greater ease than either of the instruments now in use.

Secondly. The instrument is capable of acting on one or two points at the same time, and that in every possible direction, according as the principal obstacle is found to be, as is generally the case, at one or another particular part of the pelvis.

Lastly. Although the two blunt ends are directed to be closed when first introduced, yet, when in the act of extracting, they should be always separated to a certain extent, which will effectually prevent the instrument from slipping out.

The instrument, however, will be best understood from the enclosed drawing.

Fig. 1. Represents it closed. *aa.* The two blunt ends, which are first to be introduced. *bb.* A steel collar, nicely let into the two arms of the instrument, by means of a groove in each, so as to be on a level with them. *cc.* Two indexes, which, with the blunt ends, are capable of describing a complete circle, and at the same time point out the parts on which they are acting.

Fig. 2. Represents the instrument of its proper size, and open to its utmost extent.

WILLIAM GUY.

*Chichester, June 25, 1809.*

## Figures and Tables

**Fig.1. f1:**
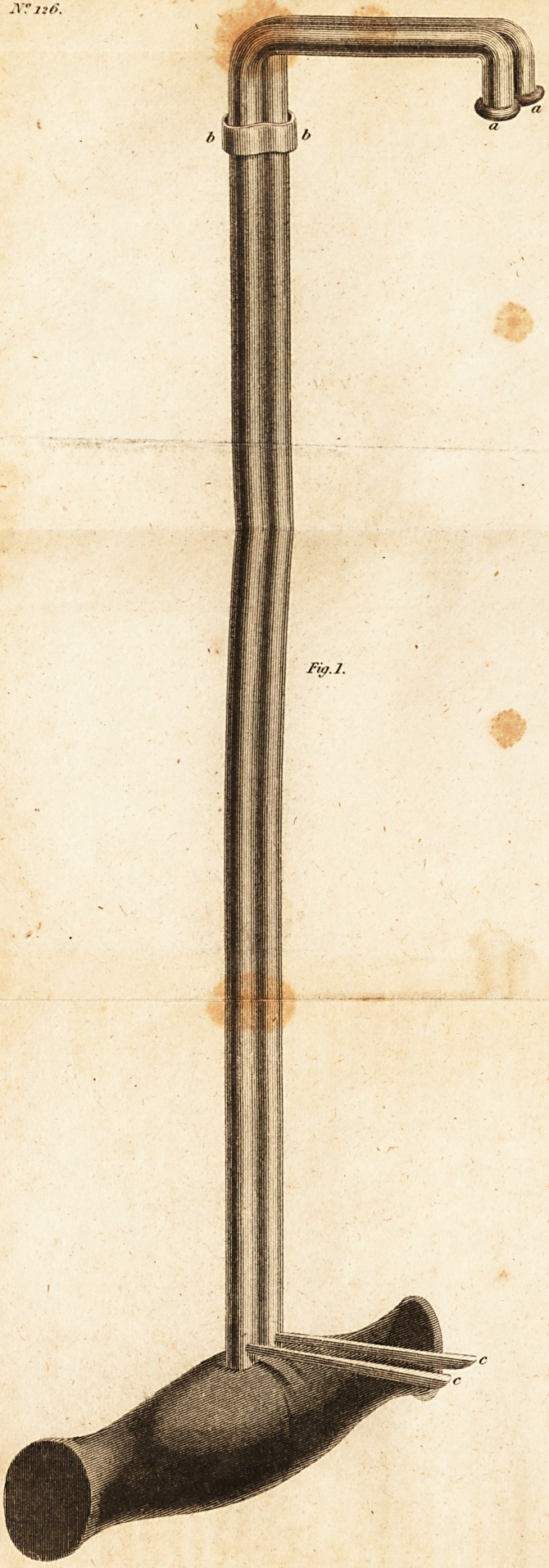


**Fig. 2. f2:**